# Quantitative molecular assessment of chimerism across tissues in marmosets and tamarins

**DOI:** 10.1186/1471-2164-13-98

**Published:** 2012-03-19

**Authors:** Carolyn G Sweeney, Elizabeth Curran, Susan V Westmoreland, Keith G Mansfield, Eric J Vallender

**Affiliations:** 1 New England Primate Research Center, Harvard Medical School, One Pine Hill Drive, Southborough, MA 01772, USA

## Abstract

**Background:**

Marmosets are playing an increasingly large and important role in biomedical research. They share genetic, anatomical, and physiological similarities with humans and other primate model species, but their smaller sizes, reproductive efficiency, and amenability to genetic manipulation offer an added practicality. While their unique biology can be exploited to provide insights into disease and function, it is also important that researchers are aware of the differences that exist between marmosets and other species. The New World monkey family Callitrichidae, containing both marmoset and tamarin species, typically produces dizygotic twins that show chimerism in the blood and other cells from the hematopoietic lineage. Recently, a study extended these findings to identify chimerism in many tissues, including somatic tissues from other lineages and germ cells. This has raised the intriguing possibility that chimerism may play an increasingly pervasive role in marmoset biology, ranging from natural behavioral implications to increased variability and complexity in biomedical studies.

**Results:**

Using a quantitative PCR based methodology, Y-chromosomes can be reliably detected in the females with male fraternal twins allowing for a relative quantification of chimerism levels between individuals and tissues. With this approach in common marmosets (*Callithrix jacchus*) and cotton-top tamarins (*Saguinus oedipus*), chimerism was detected across a broad array of tissues. Chimerism levels were significantly higher in tissues primarily derived from the hematopoietic lineage, while they were lower, though still detectable, in tissues with other origins. Interestingly, animals with a characteristic marmoset wasting disease show higher levels of chimerism in those tissues affected. Fibroblast cell lines from chimeric individuals, however, are not found to be chimeric themselves.

**Conclusion:**

Taken together, the levels of chimerism in tissues of different origins coupled with other lines of evidence suggest that indeed only hematopoietic cell lineages are chimeric in callitrichids. The chimerism detected in other tissues is likely the result of blood or lymphocytic infiltration. Using molecular methods to detect chimerism in a tissue sample seems to have allowed a substantial increase in the ability to detect these minor cell populations.

## Background

The common marmoset (*Callithrix jacchus*) has become a popular animal model in biomedical research [1]. They offer the benefits of other non-human primate model systems; however, due to their small size at adulthood (350-450 g), they harbor more reasonable husbandry needs. They reach sexual maturity at 18 months, and the females produce 3-5 offspring per year [2], with fraternal twinning the norm [3]. The fast rate of development and high level of fecundity make them a suitable model for reproductive studies. Because of their modest life span and aging-associated pathologies that parallel humans, including arthritis [4], marmosets have also shown great promise as a non-human primate model of aging and aging-related disease [5]. They have limited polymorphism at the MHC class- I and -II loci, which is believed to result in their high susceptibility to diseases [6], making them useful in studies of numerous infectious diseases. Recently, marmoset models have been developed for dengue virus infection [7], smallpox [8], and Karposi's sarcoma [9]. In neuroscience research, marmoset models have been developed recently for the study of temporal lobe epilepsy [10] as well as stroke and ischemia [11]. These join the more established models of Parkinson's disease [12], Huntington's disease [13], Alzheimer's disease [14] and multiple sclerosis [15].

The first report of a transgenic primate with germline transmission used a marmoset model [16]. This, coupled with the development of somatic cell cloned embryos [17], the ability to produce induced pluripotent stem cells [18], and the aforementioned husbandry benefits, has ensconced the marmoset as a major new player in non-human primate research going forward. Several years prior, *Callithrix jacchus *was selected as the first New World monkey genome to be sequenced. As the genome sequence becomes available, the development of molecular tools for the study of marmosets has expanded, but as these mechanisms become used more frequently in marmoset studies, the need for a greater understanding of the marmoset at a genetic level becomes more important.

Hematopoietic chimerism was first identified in Callitrichidae, the family of New World monkeys that includes marmosets and tamarins, in the 1930's [19]. This chimerism, which arises during early fetal development, occurs when the chorions of the two blastocysts fuse together, allowing for cellular exchange between them via placental anastomosis. There follows an exchange of stem cells between the dizygotic twins, resulting ultimately in the hematopoietic chimerism. During the following decades, additional studies confirmed and extended the observations, establishing chimerism across species within the family and finding chimerism in lymphatic tissues such as bone marrow, spleen, and thymus [20-23].

These studies, however, were unable to identify chimerism in other tissues not of hematopoietic origins, notably in lung or kidney [21]. This understanding, that a shared placenta during development led to hematopoietic but not other tissue chimerism, persisted until recently, when investigators using modern genetic techniques were able to detect chimerism in many somatic tissue types and even in the germ line [24]. These new results threw a wrench in the conventional theory suggesting that perhaps stem cell exchange was more prevalent in early callitrichid development than previously thought or that transdifferentiation was common. Moreover, they necessitated a reevaluation of marmoset genetic studies.

Chimerism in callitrichids was first observed by detecting discordant sex chromosomes in the karyotypes of opposite-sex twins [20,22]. Karotyping, however, was cost and time intensive and not practical for the study of large numbers of animals or tissues. Ross et al. were able to overcome this limitation by using microsatellite markers as a method of chimerism detection [24]. This allowed a much higher throughput approach and enabled a much more complete study. While both methods were successful at detecting chimeric tissue, neither one was able to quantitate the level of chimerism within an individual or within various tissue types. Quantification would allow for clarification as to whether detectable chimerism was because a tissue was truly chimeric or because it had some level of blood contamination. Karyotypic approaches would presumably be less sensitive to issues of blood contamination as tissue type is more greatly controlled, but can only interrogate few cells easily, a barrier if chimerism levels are low. The microsatellite approach allows a much deeper examination of tissues, but at the expense of certainty of tissue type.

Quantitative PCR (qPCR) can allow for the quantification of chimerism levels in tissues and allow for comparisons between chimerism levels between individuals as well as between tissues within a single individual. Following the original conceit of the karyotype studies, detecting a Y-chromosome in females with a male twin, it is possible to quantitate the amount of chimerism in a given sample. Further, since the proportion of Y-chromosome in the samples can be quantitated it is also possible to extrapolate chimerism levels in males with female twins. At the same time, by using a qPCR methodology the high-throughput advantages gained by molecular techniques remain. Many more individuals and tissues can be tested and low levels of chimerism can be confidently detected.

The marmoset is emerging as a major animal model. It combines the translational relevance and similarities to human of the rhesus macaque with the husbandry benefits and genetic tools of the mouse. One important difference, however, between the marmoset and human or any other animal model is the chimerism unique to the callitrichids. Studies of marmosets and those making use of marmoset models, especially if they are to take advantage of modern genetic techniques, necessitate a more complete and more robust understanding of marmoset chimerism; whether non-hematopoietic tissues are truly chimeric and the levels to which this chimersim may exist.

## Results and discussion

The studies that established chimerism in callitrichids used karyotype analysis to detect Y-chromosomes in females with male dizygotic twins [20,22]. Using qPCR, rather than karyotyping, it was possible to more easily test larger numbers of individuals and tissues. To detect the Y-chromosome, primers were designed to identify *SRY*. Originally referred to as the testes determining factor, *SRY *is a single copy gene located on the Y-chromosome responsible for determining the male sex in mammals. The presence of this gene in a female marmoset or tamarin therefore indicates chimeric cells gained from a male sibling in utero. In order to quantitate levels of chimerism, the prevalence of the *SRY *gene was normalized to two autosomal loci, lysozyme (*LZM*) and the vitamin D receptor (*VDR*). These two genes, again, are both single copy genes. As a control, *LZM *and *VDR *were also compared to one another across all tissues and animals without any variation (data not shown).

The metric developed for this approach, the relative normalized Y-chromosome ratio, is analogous to the Pfaffl approach in traditional mRNA-based qPCR [25]. Male animals with male twins were used to normalize all tissues tested. A relative normalized Y-chromosome ratio of one represents what might be considered a "standard" male without female chimerism while a value of zero might be considered a "standard" female without male chimerism. Males or females with opposite-sex twins would expect to show ratios of 0.5 if there was complete chimerism.

**Figure 1:**
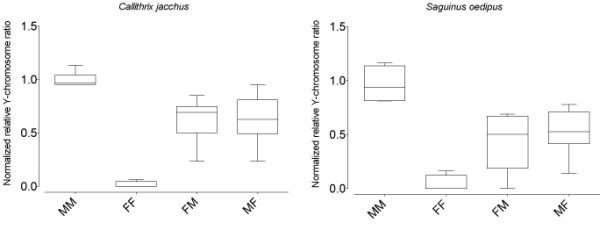
**Chimerism levels in blood**. Box and whisker plots of normalized relative Y-chromosome ratios in blood of common marmosets (*Callithrix jacchus*, left) and cotton-top tamarins (*Saguinus oedipus*, right). Animals are grouped by sex and sex of twin (MM: male with male twin, MF: male with female twin, FM: female with male twin, FF: female with female twin).

To verify this approach, the relative normalized Y-chromosome level was determined in blood for two callitrichid species, common marmoset (*Callithrix jacchus*) and cotton-top tamarin (*Saguinus oedipus*) (Figure 1). Male-male (male test individuals with male twins) ratios ranged between 0.94 and 1.13 for marmosets (n = 6) and between 0.81 and 1.16 for tamarins (n = 4). In both marmosets (n = 4) and tamarins (n = 4) female-female ratios were indistinguishable from zero except for one marmoset (0.06) and one tamarin (0.16). While a conclusive answer for these findings cannot be determined, it seems likely that the cryptic chimerism is the result of male triplets resorbed during early pregnancy, a phenomena known to occur in callitrichids [26]. This resorbtion would have allowed the male to contribute his Y-chromosome to his female littermates during the early days of uterine development, but his presence would not be detected at the time of birth.

In marmosets, male-female (male test individuals with female twins) ratios ranged from 0.24 to 0.81 (n = 10) with one outlier at 0.95, while female-male ratios ranged from 0.24 to 0.85 (n = 7). Findings were similar in tamarins, male-female ratios of 0.14-0.77 (n = 8) and female-male ratios of 0.37-0.69 (n = 7). Perhaps notable, however, was the finding of multiple female tamarins putatively with male twins that showed little to no chimerism even in blood. This finding supports earlier studies in which chimerism was not detected in all marmosets or tamarins [20,23]. It remains unclear whether this lack of hematopoietic chimerism is biologically meaningful or if it is an artifactual result of missexing of the twin, either because of misidentification of parentage or otherwise. While husbandry has greatly improved and molecular parentage and sexing methodologies have emerged that will likely ameliorate these issues going forward, these issues remain for retroactive studies.

Chimerism at the blood level varied between individuals, consistent with the results of earlier karyotypic studies [20-23]; however, direct comparison of these results to earlier works is difficult. A major limitation of the karyotypic studies is that inferences are limited to the relatively small number of cells selected for analysis, a difficulty acknowledged in those early papers. Unlike those early studies, the methodology and data presented here represent a large number of cells and as such are a better representation of the amount of chimerism in blood as a whole.

Next, tissues from 18 females with male twins (9 marmosets and 9 tamarins) were gathered and screened for the presence or absence of *SRY *and chimerism levels were quantitated. Tissues were selected based on availability and to represent lineages descended from the ectoderm (cerebrum), endoderm (liver, lung, pancreas, jejunum), and mesoderm (spleen, kidney, heart, bone marrow). Both bone marrow and spleen contain large numbers of cells from the hematopoietic lineage and are broadly classified as hematopoietic. Not all tissues were available from all animals and the pancreas, heart and bone marrow were only available from a subset of the larger group.

**Figure 2:**
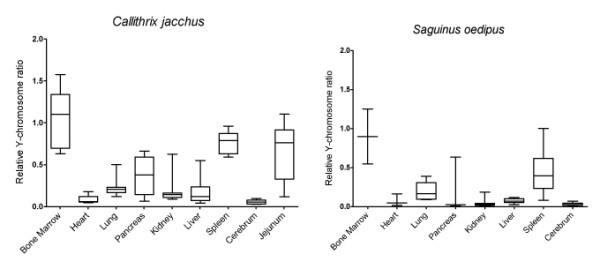
**Chimerism levels across tissues**. Box and whisker plots of normalized relative Y-chromosome ratios across tissues for common marmosets (*Callithrix jacchus*, left) and cotton-top tamarins (*Saguinus oedipus*, right). All individuals herein are females with a male twin.

Using the relative normalized Y-chromosome metric, levels of chimerism were measured across tissues (Figure 2). There were no major differences observed between patterns of chimerism in marmoset and tamarin. With the exception of one tamarin, some level of chimerism was observed in every tissue tested. The one tamarin exception never showed chimerism. The hematopoietic tissues, bone marrow and spleen, displayed high levels of chimerism significantly different (*p *< 0.001, one-way ANOVA with Bonferroni correction) from the other tissues. Those remaining tissues, while still testing positive for the presence of the Y-chromosome, showed much lower levels of chimerism statistically indistinguishable from one another. Notably for this study, there was no correlation observed between tissues derived from the same germ layer. If chimerism was driven by early stem cell transfer, it might be expected that tissues with shared lineages would show similar levels of chimerism. While this was not observed, chimerism levels were found to be correlated between tissues within an individual (*p *< 0.001, repeated measures ANOVA). These observations held when marmosets and tamarins were considered individually or together. This finding suggests a shared "basal" chimerism level for each individual.

While chimerism levels tended towards a normal distribution for most tissues, several tissues, jejunum and pancreas in particular, showed significant variation between animals and appeared to be bimodal. Notably, the same animals were consistently being observed in the high chimerism and low chimerism groups, regardless of tissue. In exploring for potential sources of this variation, it was observed that each of the animals in the high chimerism group had been diagnosed, and confirmed histologically, with chronic lymphocytic enteritis (CLE).

CLE, previously referred to as "marmoset wasting disease," is a common ailment seen in captive marmoset colonies and is characterized by a decrease in activity, muscular atrophy, and weight loss [27]. The cause (or causes) of wasting disease, CLE, is not known though several pathological features have now been identified including inflammation of the small intestine and pancreas [27]. These tissues, along with the lymphatic tissues, were the same as those in which we observed the elevated levels of chimerism. From these observations, we suspected that there was a correlation between CLE and chimerism.

**Figure 3:**
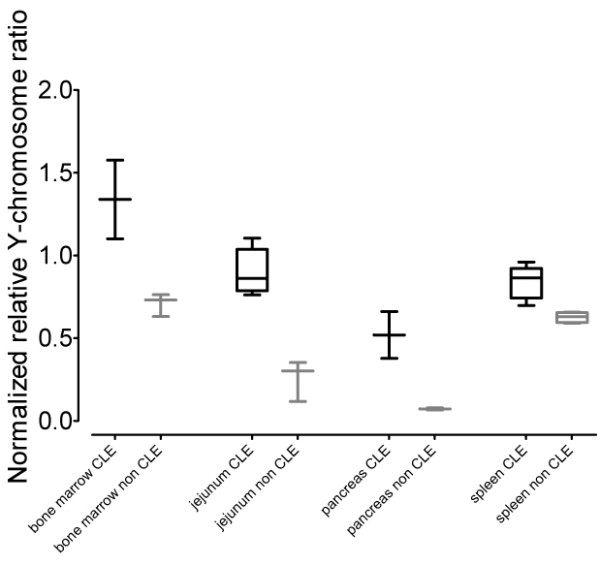
**Differential apparent chimerism in marmosets with CLE**. Box and whisker plots of normalized relative Y-chromosome ratios showing significant differences between common marmosets with or without chronic lympocytic enteritis (CLE) in bone marrow (*p *< 0.05), jejunum (*p *< 0.001), pancreas (*p *< 0.05), and spleen (*p *< 0.01).

**Figure 4:**
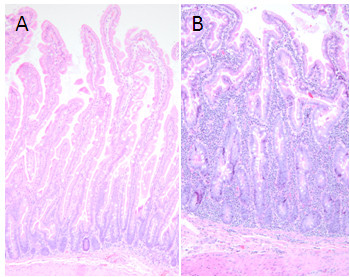
**H&E staining of marmoset intestine with or without CLE**. Small intestine from normal common marmoset (A) and marmoset affected with chronic lymphocytic enteritis (B). Note in the affected animal the blunting and fusion of villous tips accompanied by marked increased numbers of lymphocytes throughout the lamina propria (H&E stain, 10× original magnification).

Marmosets with CLE showed significantly greater levels of chimerism in jejunum (*p *< 0.001; n = 5 with CLE, n = 3 without) and pancreas (*p *< 0.05; n = 2 with CLE, n = 2 without) as well as in the bone marrow (*p *< 0.05; n = 2 with CLE, n = 3 without) and spleen (*p *< 0.01; n = 5 with CLE, n = 4 without) (Figure 3). This difference between the two groups was not apparent in any of the remaining tissues unaffected by CLE. It is hypothesized that the increase in tissue chimerism levels reflects a larger relative proportion of the chimeric lymphocytes in the tissues affected. Indeed a major histopathologic hallmark of CLE is lymphocytic infiltration in the lamina propria accompanied by villous atrophy [27]. Hematoxylin and eosin (H&E) staining shows this significant increase in the amount and number of lymphocytes in the jejunum of animals with CLE (Figure 4). While not definitive, increased chimerism in CLE-affected tissues is consistent with the hypothesis that infiltrating chimeric lymphocytes contribute to tissue chimerism, and suggest a possible interpretation for the findings of chimerism in tissues more broadly.

**Figure 5:**
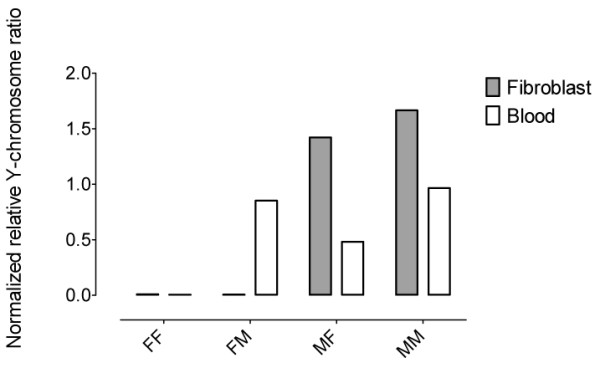
**Blood and fibroblast chimerism levels within animals**. Normalized relative Y-chromosome ratios from blood and from fibroblast cell lines derived from the same animals. Animals are shown by sex and sex of twin (MM: male with male twin, MF: male with female twin, FM: female with male twin, FF: female with female twin).

This interpretation posits that the low levels of chimerism seen in non-hematopoietic tissues result primarily from subpopulations of chimeric cells within the tissues and not from chimerism in the tissues themselves. To test this hypothesis, fibroblast cell lines were generated from skin biopsies from individuals of both sexes and chimerism levels tested (Figure 5). The fibroblast lines themselves were grown at high confluency and represented lineages from numerous individual founder cells. Blood samples from the same individuals with opposite-sex twins showed intermediate relative normalized Y-chromosome values, but as expected, fibroblasts from neither females nor males showed any evidence for chimerism. Following depletion of potential hematopoietic subpopulations during the cell culturing process, all evidence of chimerism was lost.

## Conclusion

Callitrichid primates, especially common marmosets, are becoming increasingly important as a model system for many human diseases. As their translational relevance grows, an appreciation and understanding of their unique biology becomes more necessary. One primary factor in that unique biology is the chimerism that arises between twins. Alone, hematopoietic chimerism can have important impacts on studies. Associations of any kind relying of genotyping need to be aware of the source of their genomic DNA and the potential confounds that chimeric blood may introduce. Similarly, studies focused on immune response would be wise to consider the potential effect of chimeric lymphocytes. While these confounds have been known for quite some time, their importance has grown immensely in the post-genomic era.

The primary question to be addressed here was the finding, raised by Ross et al. [24], regarding chimerism outside of the hematopoietic system. Here, using a novel molecular methodology built around quantitative PCR, chimerism was found in all organ systems tested. These levels of chimerism varied greatly, however, and largely did not reach the levels seen in blood, bone marrow, or other hematopoietic tissues. Further, levels of chimerism, while correlated within individuals, did not vary in tissues along germ layer lines suggesting that the cause of chimerism in these tissues in adults was not an early development phenomenon. Given the generally low levels of chimerism in tissues, a reasonable hypothesis is that blood or lymphocytic infiltration is driving the apparent chimerism seen in the organ systems.

This hypothesis was supported by the finding that animals with a pervasive disease, chronic lymphocytic enteritis, show higher levels of chimerism in affected tissues, a finding consistent with increased concentrations of chimeric lymphocytes. Further, studies of pure fibroblasts, uncontaminated by cells of hematopoietic origin, do not show evidence for chimerism. These findings are also in line with historical understandings of the origin of chimerism that a shared blood flow, presumably including hematopoietic stem cells, occurs between twins during uterine development. This hypothesis reconciles the observation of chimerism in organs with the mechanism from which chimerism in believed to be derived. In this case, it would appear that novel molecular mechanisms have allowed researchers to identify a small subpopulation of chimeric cells within a larger non-chimeric organ. In binary experiments, it is then easy to misattribute this chimerism to the organ proper.

What remains unexplained are the findings by Ross et al. of chimerism in sperm and transmission of chimerically-derived alleles across generations [24]. The studies here cannot address these questions. As primordial germ cells are a lineage unto themselves it is possible that their development is sufficiently different as to represent a new source of chimerism. This seems difficult to reconcile with early developmental biology, however. It is also possible that indeed the chimerism observed in somatic tissues is true chimerism, though multiple lines of evidence suggest this too is unlikely. Ultimately, what will be required to answer these questions thoroughly are greater genetic and physical resources, additional genetic variation that can be used to distinguish with confidence chimeric cells and a refinement of the tissues and cell types that can be studied. In the meantime, however, it can be enough to state with confidence that chimerism, even if only from blood and lymphocytes, can be found in any tissue or organ at low levels in modern molecular studies.

Callitrichid hematopoietic chimerism is a unique and unusual character among mammals. It has implications both for the animals themselves as well as for those researchers using them as model systems. This chimerism, however, does not seem to extend beyond the hematopoietic system. These together define the boundaries for study, both regarding the implications of chimerism as well as its ultimate molecular and evolutionary origins.

## Methods

### Animals

All animal blood and tissue samples were derived from captive born animals from colonies, common marmoset (*Callithrix jacchus*) and cotton-top tamarin (*Saguinus oedipus*), at the New England Primate Research Center. Contemporaneous birth records were used to identify the sex of the animal and other shared-birth siblings. Most marmoset and tamarin births at the NEPRC are twins (46.0% of *Callithrix jacchus*, 57.7% for *Saguinus oedipus*) or triplets (37.0% for *Callithrix jacchus*, 14.3% for *Saguinus oedipus*). Occasionally stillborn siblings, often in triplets or other larger multiple births, will be unsexed. Mixed-sex twins and triplets were used for comparing chimerism in blood and tissues. Only females with a male twin or triplet were chosen for comparing chimerism in tissues. While using females from twin births only would have allowed for greater analytical ease, triplet births were included to help increase the sample size. Also, it should be noted that cryptic siblings resorbed early during development may affect overall chimerism levels. DNA from the blood of males with male twins was used as a standard in qPCR analysis, and DNA from blood of females with female twins was used as a negative control. In animals with chronic lymphocytic enteritis, confirmation was based on histopathology of intestinal samples with characteristic villus blunting and expansion of the lamina propia with lymphoplasmacytic infiltrates. All animals were maintained in accordance with the guidelines of the Harvard Medical School Standing Committee on Animals and the Guide for Care and Use of Laboratory Animals of the Institute of Laboratory Animal Resources, National Research Council. The New England Primate Research Center is accredited by the American Association for the Accreditation of Laboratory Animal Care.

### Tissue and blood collection

Tissue samples were collected immediately postmortem from animals euthanized following unrelated procedures or in the course of normal end-of-life palliative care. Tissue samples were immediately flash frozen and stored at -80°C until use. DNA was extracted using the Maxwell 16 system (Promega Corporation, Madison, WI) for cerebrum, spleen, liver, pancreas, kidney, lung, bone marrow, jejunum, or using the DNeasy kit (Qiagen, Valencia, CA) for heart samples. 1-3 mL venous blood was collected from living animals in EDTA-vacutainer tubes, and DNA was extracted using the Flexigene kit (Qiagen, Valencia, CA) following manufacturer protocols. The extracted DNA was quantified and diluted to 50 ng/μL for standard PCR amplification and 10 ng/μL for qPCR.

### Fibroblast line creation

Skin biopsies were obtained from four marmosets (two male and two female) from the NEPRC colony. The samples were trimmed into smaller pieces of approximately one mm^2 ^in size and placed in tissue culture plates. They were then incubated at room temperature until becoming adherent. The samples were cultured with Dulbecco's modified Eagle's medium (DMEM) with penicillin-streptomycin (55 μg/mL) fetal bovine serum (10%), and 100 μM non-essential amino acids. The resulting fibroblasts were allowed to grow out for five passages before being harvested for genomic DNA extraction as above.

### PCR amplification

DNA was PCR amplified using markers for *SRY *(F- TTGCTTACTGAAGCCGAAAAA, R - TGCATGGCCTGTAGTTTCTG), *LZM *(F- GGCCAAATGGGAGAGTGAT, R - TGAAATATCCCATAATCAGRGCTTT), and *VDR *(F- GGAGGCCTTGAAGGACAGT, R- CCGGAACTGGCAGAAGTC). PCR products were run on the QIAxcel System (Qiagen, Valencia, CA) to determine the presence or absence of *SRY *in the sample source. *SRY *levels were quantitated using the Roche Universal ProbeLibrary System on the Roche LightCycler 480 (Roche Applied Science, Indianapolis, IN). Each 20 μL reaction included 5 μL DNA at 10 ng/μL, 4 μL H_2_O, 0.8 μL primer, 0.2 μL probe (*SRY*: #79, *LZM*: #80, *VDR*: #68), and 10 μL Roche LightCycler 480 Mastermix. The ratio of *SRY *to the reference gene was calculated based on the efficiencies of the two reactions and a control sample of a male with a male twin.

$$Ratio=\left[{\left(E_{SRY}\right)}^{\Delta CP}SRY\right]/\left[{\left(E_{Ref}\right)}^{\Delta CP}Ref\right]\;\left[25\right].$$

Efficiencies for the primer sets were calculated using serial dilution curves. Each reaction was performed in triplicate and final values were based off the average of the three replicates. Positive controls were also employed comparing the two autosomal loci, *LZM *and *VDR*.

## Competing interests

The authors declare that they have no competing interests.

## Authors' contributions

EJV conceived of the study. EC and SWV obtained the tissue samples. CGS performed the molecular studies. KGM assisted with CLE studies. EJV and CGS analyzed the data. All authors read and approved the final manuscript.
